# Induction of kidney tumours by a single dose of dimethylnitrosamine: dose response and influence of diet and benzo(a)pyrene pretreatment.

**DOI:** 10.1038/bjc.1980.41

**Published:** 1980-02

**Authors:** P. F. Swann, D. G. Kaufman, P. N. Magee, R. Mace

## Abstract

Seven days on a protein-free diet increases the susceptibility of rats to the action of DMN as a renal carcinogen. The dose response for the induction of kidney tumours by a single dose of dimethylnitrosamine (DMN) in these rats is reported. The first tumour was not found until 28 weeks after the dose. At 100 weeks the incidence ranged from 22.5% at the lowest dose (20 mg/kg) to 97% at the highest dose (60 mg/kg). The incidence in probits at any time between 50 and 100 weeks was linearly related to the log dose. Epithelial and mesenchymal tumours were produced in an approximate ratio of 2:1. The protein-free diet alters the rate of metabolism of DMN in the rat, and increases the alkylation of nucleic acids by this carcinogen in the kidney. Further treatment of the rat with benzo(a)pyrene can reverse, to some extent, the change in metabolism, but does not reverse the change in alkylation. It is shown that the change in kidney-tumour incidence produced by the change in diet, and by the treatment with benzo(a)pyrene, corresponds to the changes these treatments produce in the alkylation of kidney DNA by the carcinogen.


*Br. J. Cancer* (1980) **41**, 285

### INDUCTION OF KIDNEY TUMOURS BY A SINGLE DOSE OF DIMETHYLNITROSAMINE: DOSE RESPONSE AND INFLUENCE OF DIET AND BENZO(A)PYRENE PRETREATMENT

P. F. SWANN, D. G. KAUFMAN*, P. N. MAGEE† AND R. MACE

*From the Courtauld Institute of Biochemistry, The Middlesex Hospital Medical School, London W1P 7PN, and the *Department of Pathology, School of Medicine, University of North Carolina, Chapel Hill, N.C. 27514, U.S.A.*

Received 30 May 1979 Accepted 12 October 1979

**Summary.—Seven days on a protein-free diet increases the susceptibility of rats to the action of DMN as a renal carcinogen. The dose response for the induction of kidney tumours by a single dose of dimethylnitrosamine (DMN) in these rats is reported. The first tumour was not found until 28 weeks after the dose. At 100 weeks the incidence ranged from 22·5% at the lowest dose (20 mg/kg) to 97% at the highest dose (60 mg/kg). The incidence in probits at any time between 50 and 100 weeks was linearly related to the log dose. Epithelial and mesenchymal tumours were produced in an approximate ratio of 2:1. The protein-free diet alters the rate of metabolism of DMN in the rat, and increases the alkylation of nucleic acids by this carcinogen in the kidney. Further treatment of the rat with benzo(a)pyrene can reverse, to some extent, the change in metabolism, but does not reverse the change in alkylation. It is shown that the change in kidney-tumour incidence produced by the change in diet, and by the treatment with benzo(a)pyrene, corresponds to the changes these treatments produce in the alkylation of kidney DNA by the carcinogen.**

THE DISCOVERY that only a single dose of dimethylnitrosamine (DMN) is needed to induce kidney tumours in the rat (Magee & Barnes, 1962) was soon followed by the report that some other N-nitroso compounds are equally effective (Druckrey *et al.*, 1964). The ability to produce cancer with a single dose of carcinogen opened the way to a number of previously impossible experiments. However, each of these carcinogenic nitroso compounds had some disadvantage which reduced its usefulness as an experimental tool. Some produced tumours in many organs so that it was difficult to study one single type of tumour, and others, which were specific for just one organ, required a very large dose to induce even a low incidence of tumours. For example a median lethal dose of DMN produced only a 20% incidence of kidney tumours. This low incidence of tumours, and the many severe changes in the metabolism of the animal produced by the near lethal dose, limited the usefulness of this means of inducing cancer. However, kidney tumours can be induced in every rat if the animals are given a protein-free diet for 7 days before, and 5 days after, the single dose of DMN (Swann & McLean, 1968; Hard & Butler, 1970; McLean & Magee, 1970). The diet influences the effectiveness of the nitrosamine as a renal carcinogen in 2 ways: it allows the rat to tolerate a much larger dose (the median lethal dose is almost doubled) (McLean & Verschuuren, 1969); and it alters the pharmacokinetics of the nitrosamine so that the proportion of any dose activated to the proximal carcinogen in the kidney is greatly increased (Swann & McLean,

† Present address: Fels Research Institute, Temple University School of Medicine, Philadelphia, Pa 19140, U.S.A.

1971). These 2 effects complement each other so that a dose of DMN well below the $LD_{50}$ will induce kidney tumours in every one of the rats (Swann & McLean, 1968; Hard & Butler, 1970; McLean & Magee, 1970).

The histogenesis and histological development of these kidney tumours has been studied (Hard & Butler, 1970, 1971) and an attempt has been made to identify the biochemical events crucial to their development, by studying the effect on them of inhibitors of protein and nucleic acid synthesis (Stewart & Magee, 1973). Because the results of this study could not be compared with a dose–response curve it was unsatisfactory. It could not show whether a large or a small change in dose of carcinogen was needed to produce the same change in incidence as produced by the drug treatment. Furthermore, a dose–response curve would have indicated the optimum number of animals, and the most appropriate dose of carcinogen.

In this paper the dose–response for the induction of kidney tumours by DMN in these protein-deprived rats is described, and it is shown that the effect of the change of diet, and of pretreatment with benzo(a)pyrene, on the incidence of kidney tumours produced by DMN corresponds to the effect of these treatments on the alkylation of kidney DNA by the nitrosamine.

### MATERIALS AND METHODS

*Animals.*—Wistar-derived male rats of CFN stock (5–6 weeks old; 130–140 g) were bought from Carworth Farms, New City, N.Y., U.S.A. After 5–7 days' acclimatization, during which they were fed Purina Chow (Ralston Purina Corp., St Louis, Mo., U.S.A.), they were changed to a semi-synthetic diet containing no protein (McLean & McLean, 1966). Those rats to be treated were weighed and given an i.p. injection of a solution of DMN in 0·15M NaCl (0·5 ml/100 g body wt) on the morning of the 7th day on the diet. (Controls were injected with a similar volume of 0·15M NaCl at the same time.) After a further 5 days on this diet (*i.e.* a total of 12 days) the rats were returned to the normal diet of Purina Chow. While they were receiving the protein-deficient diet the rats were kept in cages with gridded bottoms so that they were unable to eat faeces or bedding.

Outbred Osborne Mendel rats and inbred Buffalo rats were supplied by the Laboratory Aids Branch, National Institutes of Health, Bethesda, Md 20014, U.S.A.

*Determination of the effect of a protein-free diet on the $LD_{50}$ of DMN.*—The $LD_{50}$s were determined (Weil, 1952) in rats on a normal diet (Purina Chow) or on protein-free diet. Twenty-four CFN male rats, 24 Osborne Mendel male rats, and 24 male Buffalo rats (in all cases 140–170 g) were acclimatized, kept on a protein-free diet, and treated with DMN as detailed above. The $LD_{50}$ in these rats was compared with that in a similar number of rats fed only Purina Chow.

*The influence of the protein-deficient diet or pretreatment with benzo(a)pyrene on the metabolismof DMN by liver and kidney slices* in vitro, *and on the methylation of liver and kidney DNA* in vivo *by a single dose of* [$^{14}$C]DMN.—Previously described methods (Swann & McLean, 1971) were used to assess the influence of diet and pretreatment with benzo(a)pyrene on the extent of methylation of liver and kidney DNA produced *in vivo* by a dose of DMN, and upon the ability of liver and kidney slices to metabolize DMN *in vitro*. In both studies CFN rats were fed either a normal diet, or a protein-free diet for 7 days. After 4 days, half the rats on each diet were given a single i.p. dose of benzo(a)pyrene (20 mg/kg body wt as an 8mg/ml solution in maize oil). On the 7th day 5 animals from each group were given a single i.p. dose of [$^{14}$C] DMN (40 mg/kg body wt). The animals on the normal diet were killed 8 h, and those on the protein-free diet 15 h, after the dose, and the amount of N-7-methylguanine in the DNA of the pooled kidneys measured. The interval between dose and death was longer (15 h) in the animals on the protein-free diet because these animals metabolize DMN more slowly. The remaining animals were also killed and the ability of slices of their livers and kidneys to metabolize DMN *in vitro* was measured.

*Determination of the incidence of kidney tumours produced by various amounts of DMN, and the effect on that incidence of pretreatment with benzo(a)pyrene.*—The CFN rats were acclimatized, changed to a protein-free diet and treated with DMN (20, 30, 40, 50 or 60 mg/kg body wt) as detailed above. The number of rats in each group is given in Table II. There was one untreated control group of rats on a normal diet (Purina Chow), and another on the protein-deficient diet. Both groups were injected with 0·15M NaCl (0·5 ml/100 g body wt).

The effect of benzo(a)pyrene on DMN-induced kidney-tumour incidence in the protein-deprived rat was studied by giving one dose (20 mg/kg body wt i.p. as 8 mg/ml solution in maize oil) 3 days before DMN (40 mg/kg body wt). One control group was given only benzo(a)pyrene, and one was kept on a normal diet (Purina) and given a single dose of DMN (40 mg/kg body wt).

Animals were killed if moribund or found to have a palpable tumour. All animals were necropsied. The liver, kidneys, brain, lungs and testes of every rat, and in most cases the spleen, pancreas and bladder, were taken for histological examination. Tissues were fixed in neutral formalin, and the histological preparations stained with haematoxylin and eosin.

*Chemicals.* — Dimethylnitrosamine (Eastman Kodak, Rochester, N.Y., U.S.A.) was redistilled before use. Benzo(a)pyrene from the same source was used without further purification. [$^{14}$C]Dimethyl-nitrosamine (54 mCi/mmol) was synthesized from [$^{14}$C]-dimethylamine (Radiochemicals Centre, Amersham, U.K.) by the method of Dutton & Heath (1956) and diluted with DMN as required.

### RESULTS

In the CFN rats the $LD_{50}$, which had been 35·7 mg/kg body wt (log standard deviation 0·0685, *i.e.* between 30·4 and 41·8 mg/kg) in rats fed Purina Chow, was increased to 60·7 mg/kg body wt (log s.d. 0·0290, *i.e.* between 56·7 and 65 mg/kg) by 7 days' pre-treatment with a protein-free diet. The protein-deficient diet also protected Osborne Mendel rats, increasing the $LD_{50}$ from 31·6 mg/kg (29·4–34) on the normal diet, to 46 mg/kg (41·3–51·8), but did not protect Buffalo rats, where the $LD_{50}$ which was 51·6 mg/kg (47·3–55·9) on the normal diet, actually decreased to 45·2 mg/kg (43·2–47·3) when the rats were pretreated with the protein-free diet.

A single dose of 40 mg DMN/kg body wt produced 0·7 mmol N-7-methylguanine/mol guanine in the DNA of the pooled kidneys of 5 CFN rats on a normal diet; 1·2 mmol in the kidneys of 5 rats on a protein-free diet; and 1·2 mmol in the kidneys of 5 rats pre-treated with benzo-(a)pyrene while on a protein-free diet. In this experiment the standard deviation of these numbers was not measured, but in our experience the variation from rat to rat was small. In another experiment the alkylation of liver DNA in 22 separate rats given DMN had an s.d. of 12%. The variation in the alkylation of kidney DNA has not been studied so thoroughly but appears to be similar.

Liver slices *in vitro* were able to metabolize DMN to $CO_2$ at the following rates (nmol $CO_2$ produced/g tissue/h): liver slices from CFN rats on a normal diet, 361 ± 29; liver slices from CFN rats after

TABLE I.—*The mortality among rats treated with dimethylnitrosamine (DMN)*

| Group | Treatment: DMN (mg/kg) | Treatment: Diet | No. rats treated | No. dying in first 24 weeks (%) | No. dying 25–104 weeks (%) | Survivors at 104 weeks (%) |
|---|---|---|---|---|---|---|
| 1 | 40 | Purina | 60 | 16 (27) | 32 (53) | 12 (20) |
| 2 | — | Purina | 25 | 0 | 10 (40) | 15 (60) |
| 3 | 60 | Protein-free | 55 | 20 (36) | 34 (62) | 1 (2) |
| 4 | 50 | Protein-free | 45 | 7 (16) | 37 (82) | 1 (2) |
| 5 | 40 | Protein-free | 55 | 13 (24) | 42 (76) | 0 |
| 6 | 30 | Protein-free | 40 | 1 (2·5) | 32 (80) | 7 (17·5) |
| 7 | 20 | Protein-free | 40 | 0 | 20 (50) | 20 (50) |
| 8 | — | Protein-free | 25 | 0 | 6 (24) | 19 (76) |
| 9 | — | Protein-free + BP* | 25 | 1 (4) | 11 (44) | 13 (52) |
| 10 | 40 | Protein-free + BP* | 99 | 55 (55) | 42 (42) | 2 (2) |

* 20 mg benzo(a)pyrene/kg body wt.

FIG. 1.—The appearance of kidney tumours after a single dose of DMN to rats temporarily on a protein-free diet. 60 mg/kg body wt ○—○; 50 mg/kg ■—■; 40 mg/kg . — .; 30 mg/kg △—△; 20 mg/kg ●—●. Each point represents the time at which one rat was found to have a kidney tumour.

FIG. 1(a).—Fig. 1 on which is superimposed the appearance of kidney tumours in rats given 40 mg DMN/kg after pretreatment with 20 mg benzo(a)pyrene/kg. (Both treatments while the rats were temporarily on a protein-free diet.)

FIG. 1(b).—Fig. 1 on which is superimposed the appearance of kidney tumours in rats given 40 mg DMN/kg while on a *normal* diet.

7 days on protein-free diet, 90 ± 11; liver slices from CFN rats after 7 days on protein-free diet and with benzo(a)pyrene treatment at 4 days, 283 ± 28. Kidney slices from CFN rats were able to metabolize DMN to $CO_2$ at the following rates (same units as for liver): kidney slices from rats on a normal diet, 29·7 ± 1·3; kidney slices from rats after 7 days on a protein-free diet, 16·5 ± 1·0; kidney slices from rats after 7 days on a protein-free diet but with benzo(a)pyrene treatment at 4 days, 34·4 ± 3·0.

The effect of dose on the incidence of kidney tumours was studied in CFN rats given either 20, 30, 40, 50 or 60 mg DMN/kg body wt while on the protein-free diet. The number of rats in each group is given in Table I and details of their treatment in the Materials and Methods section.

Of the 499 rats at the beginning of the experiment, 390 were alive when the first kidney tumour was found. Most of the deaths (107/109) before the first kidney tumour was found (*i.e.* in the first 27 weeks) were within the first 2 weeks of the treatment, as a result of the acute toxic effects of the nitrosamine. 55 (55%) were in the group pretreated with benzo(a)pyrene, in comparison with a mortality of 24% in the group treated with DMN alone (Table I, Group 5). A possible explanation for this is in the Discussion.

There were very few deaths from natural causes (infections, etc.) during the course of the experiment, and the number of animals lost in this way would be too small to distort the figures for tumour incidence materially. For example in the rats given DMN while on the protein-free diet (Groups 3–7) most of the tumours were found between 25 and 75 weeks after the dose. During this period 126 rats died with kidney tumours; only 10 from other causes.

The time of appearance of kidney tumours in the rats treated with DMN while on the protein-free diet (Groups 3–7) is shown in Fig. 1. These points represent the time the rat died and was subsequently found to have a kidney tumour, or the time at which the rat was killed after a kidney tumour had been detected by palpation. If the tumour incidence in Fig. 1 at any one time between 50 and 100 weeks is plotted as incidence *vs* dose, a sigmoid curve is obtained (*e.g.* Fig. 2(a), which shows the results at 60 weeks). These curves can be converted into straight lines by replotting them as probit-of-incidence against log dose (for example Fig. 2(b)), a maximum-likelihood regression analysis can be carried out, and fiducial lines calculated (Finney, 1971). Regression analysis was carried out on the results in Fig. 1 at each 10-week interval between 50 and 100 weeks. The incidence in probits ($Y$) can be expressed as a function, $Y = bx + c$ (where $x$ is the log dose, and $b$ and $c$ constants), and the standard error of $b$ and the confidence limits for the position of the line (*i.e.* the standard error of the probit $\bar{Y}$ at the weighted median dose $\bar{x}$) calculated. The results were: 50 weeks $b = 5{\cdot}46 \pm 0{\cdot}75$, $c = -3{\cdot}95$, $\bar{Y} = 4{\cdot}87 \pm 0{\cdot}10$; 60 weeks $b = 5{\cdot}65 \pm 0{\cdot}72$, $c = -3{\cdot}95$, $\bar{Y} = 5{\cdot}08 \pm 0{\cdot}10$; 70 weeks $b = 6{\cdot}22 \pm 0{\cdot}81$, $c = -4{\cdot}48$, $\bar{Y} = 5{\cdot}30 \pm 0{\cdot}10$; 80 weeks $b = 7{\cdot}07 \pm 0{\cdot}84$, $c = -5{\cdot}54$, $\bar{Y} = 5{\cdot}38 \pm 0{\cdot}11$; 90 weeks $b = 6{\cdot}16 \pm 0{\cdot}79$, $c = -4{\cdot}03$; $\bar{Y} = 5{\cdot}36 \pm 0{\cdot}11$; 100 weeks $b = 5{\cdot}52 \pm 0{\cdot}75$; $c = -2{\cdot}84$; $\bar{Y} = 5{\cdot}53 \pm 0{\cdot}11$.

The time of appearance of kidney tumours in the rats treated with DMN and with benzo(a)pyrene is given in Fig. 1(a), and in those given DMN on a normal diet in Fig. 1(b). In each of these figures the

FIG. 2.—The dose response for the induction of kidney tumours 60 weeks after a single dose of DMN given while the rats were temporarily on a protein-free diet. (A) The results are plotted as incidence *vs* dose (B) they are plotted as probit of incidence *vs* log dose, showing both the calculated regression line and the 95% fiducial limits.

results from Fig. 1 are also given to facilitate comparison.

The total number of tumours of the kidney, lung and other organs is given in Table II. Of the 85 rats not receiving DMN, only one had a tumour in the kidney, and this appeared to be a lipoma. The kidney tumours in the DMN-treated rats were either mesenchymal or epithelial. Several rats had tumours of both kinds in one kidney. It is difficult to estimate the exact proportion of tumours which were epithelial or mesenchymal, since the rapidly growing mesenchymal tumours tended to dominate the histology whenever they were present, but there seemed to be twice as many epithelial as mesenchymal tumours (Table III). Seventy-two of the rats given DMN had lung tumours, either adenomas or adenocarcinomas. No lung tumours were found in the untreated rats.

It is reasonable to ascribe the kidney and lung tumours to the nitrosamine treatment, though some other types of tumours were found in both treated and untreated animals. The majority of these were fibrosarcomas at several sites, mostly just below the skin. Some apparently neoplastic lesions were also found in the livers; most appeared to be hepatomas, with a few angiomas and cholangiomas. Although the incidence of these lesions was higher in the nitrosamine-treated

TABLE II.—*Tumours in rats treated with DMN*

| Group | Treatment: DMN (mg/kg) | Treatment: Diet | No. rats† | No. with kidney tumours (%) | No. with lung tumours (%) | Other tumours |
|---|---|---|---|---|---|---|
| 1 | 40 | Purina | 44 | 22 (50) | 14 (32) | Liver (7); skin (1); fibrosarcoma possibly of stomach (1) |
| 2 | — | Purina | 25 | 1 (4) | 0 | Brain (1); pancreas (1); chondrosarcoma (1); fibrosarcoma (3) |
| 3 | 60 | Protein-free | 35 | 34 (97) | 7 (28) | Fibrosarcmoa (1); liver (1) |
| 4 | 50 | Protein-free | 38 | 36 (95) | 7 (18) | Fibrosarcoma (1); liver (3); pancreas (1) |
| 5 | 40 | Protein-free | 42 | 38 (90) | 6 (14) | Liver (2); pituitary (1) |
| 6 | 30 | Protein-free | 39 | 30 (77) | 12 (31) | Liver (4) |
| 7 | 20 | Protein-free | 40 | 13 (32) | 8 (20) | Liver (3) |
| 8 | — | Protein-free | 25 | 0 | 0 | Subcutaneous squamous carcinoma (1); liver (3) |
| 9 | — | Protein-free + BP* | 24 | 0 | 0 | Liposarcoma (1); fibrosarcoma (3); pituitary (1); bladder (1) |
| 10 | 40 | Protein-free + BP* | 44 | 35 (75) | 16 (36) | Liver (1); fibrosarcoma (3); bladder (1); testis (1) |

* 20 mg benzo(a)pyrene/kg body wt.
† No. rats surviving 25 weeks or more after the dose.

TABLE III.—*Histological type of kidney tumour induced by a single dose of DMN*

| Group | Treatment: DMN (mg/kg) | Treatment: Diet | No. rats with kidney tumours | No. with only epithelial tumours (%) | No. with only mesenchymal tumours (%) | No. with both mesenchymal and epithelial tumours (%) |
|---|---|---|---|---|---|---|
| 1 | 40 | Purina | 21 | 18 (86) | 3 (14) | 0 |
| 3 | 60 | Protein-free | 34 | 11 (32) | 6 (18) | 17 (50) |
| 4 | 50 | Protein-free | 36 | 21 (58) | 5 (14) | 10 (28) |
| 5 | 40 | Protein-free | 38 | 19 (50) | 8 (21) | 11 (29) |
| 6 | 30 | Protein-free | 30 | 23 (77) | 3 (10) | 4 (13) |
| 7 | 20 | Protein-free | 13 | 9 (69) | 4 (31) | 0 |
| 10 | 40 | Protein-free + BP* | 31 | 24 (77) | 2 (6) | 5 (16) |

Abbreviations: DMN, dimethylnitrosamine; BP, benzo(a)pyrene.
* 20 mg benzo(a)pyrene/kg.

animals, it is not certain that they were caused by the nitrosamine treatment: the incidence of the lesions was not dose-related, and it is generally accepted that a single dose of DMN does not induce liver tumours (Craddock, 1976).

### DISCUSSION

Before the strain of rats for the main experiments was chosen the effect of the protein-free diet on the toxicity of DMN was measured in 3 strains. It was found that 7 days on the diet reduced the susceptibility of CFN and Osborne Mendel rats to the toxic effects of DMN, but did not protect the Buffalo rat. Because of this CFN rats were chosen for all the subsequent experiments.

The Buffalo is not the only strain in which the protein-free diet has no effect on the toxicity of the nitrosamine (Waynforth *et al.*, 1977). The most striking effect of DMN-poisoning is liver damage, probably induced by the reactive metabolites of the nitrosamine. The protein-free diet decreases the ability of the rat to metabolize DMN. The liver is affected to a greater extent than other organs, and it is believed that this may be the basis for the protection (Swann & McLean, 1971). However, Waynforth *et al.* (1977) found that the protein-free diet decreased the rate of metabolism in the unprotected as well as the protected strains. It is not known, therefore, why there was a different effect on toxicity in different strains. The explanation may be that liver damage is not the only determinant of death from DMN poisoning. Effusion into the pleural cavity and bleeding into the gut are often seen at necropsy of the poisoned animals (Barnes & Magee, 1954). A counterpart to the decrease in the capability of the liver to metabolize DMN is an increase in the amount metabolized in other organs (Swann & McLean, 1971) which increases the damage in these organs. It is possible that in some strains of rat any protective effect of diet is counterbalanced by an increase in the contribution of pathological changes in the lungs and other organs to the death of the animals.

The time of appearance of kidney tumours in rats given a single dose of DMN is given in Fig. 1. In most cases this was the time that the tumours were of sufficient size to be detected by palpation, or to kill the animal. The first tumour was found 28 weeks after administration of the carcinogen. It is particularly interesting that some tumours, mostly small adenomas, were not found until the surviving rats were killed 104 weeks after the carcinogen had been administered, and microscopic examination of the kidneys was carried out. It is difficult to assess the significance of these lesions, but the steady increase in the number of clinically significant tumours in the groups given the lower doses (Fig. 1) throughout the period from 30 to 104 weeks, suggests that, given sufficient time, many would have progressed to cancer. This means that one cannot speak of an absolute incidence of tumours, only of the observed incidence at a given time.

The first tumour was found at 32 weeks in the rats given 60 mg DMN/kg and at 28, 39, 43, and 35 weeks in those given 50, 40, 30, and 20 mg/kg. The lack of a relationship between dose and the time of appearance of the first tumour is in strong contrast to the effect of a single dose of benzo(rst)pentaphene on the hamster cheek pouch (Wodinsky *et al.*, 1965) where the first tumour appeared much sooner after a large dose than after a small one. Although the time to appearance of the first tumour was not influenced by the dose, the average time between the dose and appearance of each tumour was much greater in the rats given the lower doses than in those given the higher doses (Fig. 1). A relationship between dose and the time of appearance of tumours has also been found when brain tumours were induced in the offspring of rats given a single dose of ethylnitrosourea (Druckrey *et al.*, 1970; Swenberg *et al.*, 1972) and in many experiments where carcinogens were given continuously (Druckrey, 1967). There was no significant difference between the time of appearance of the mesenchymal and epithelial tumours.

It is not known why the first tumour takes so long to appear, but it is probably not simply the time needed for the tumour to grow to a sufficient size to be detected. Microscopic study (Hard & Butler, 1971) has shown that altered cells, which seem to be precursors of the tumour, can be seen in the kidney only a few days after the dose. Yet tumours are not found, even with the microscope, until 16 weeks or more have passed. Similarly, cells taken into tissue culture from the kidneys of these rats 24 h after they have been given the nitrosamine have altered growth characteristics, but do not transform into fully malignant cells until after several passages (Hard & Borland, 1977; Hard, 1978). Other experiments in tissue culture have also shown that time is essential for the full development of malignancy (Laerum & Rajewsky, 1975; Barrett & Ts'o, 1978; Roscoe & Claisse, 1978) and are in general accord with conclusions drawn from pathology (Foulds, 1969) and mathematical analysis of the relationship between age and the incidence of cancer in man (Doll,

1971; Peto, 1977) that transformation of tissue from the normal state to cancer is a multistage process. The increase in the average time for the development of tumours (Fig. 1) as the dose is lowered suggests the interesting possibility that the initiation of cancer is not just a switch from normality to the first premalignant stage, but a process which propels the cell towards the malignant state, and that the time needed to achieve full malignancy depends upon the intensity of that initial propulsion, *i.e.* the nature and dose of carcinogen.

Previous studies (Swann & McLean, 1971) had shown that pre-treatment with the protein-free diet altered the pharmacokinetics of the nitrosamine so that a greater proportion of any dose was activated to the proximal carcinogen in the kidney. When designing these experiments we hoped to discover whether the increase in tumour incidence was entirely the result of the change in the pharmacokinetics or whether the diet also produced a change in the inherent susceptibility of the kidney. To decide this it was necessary to find the difference in incidence of tumours when DMN is given to rats on a protein-free diet, and on a normal diet; and to compare this with the difference predicted from knowledge of the pharmacokinetics. If there was no difference between the observed and predicted differences, there must be no change in the inherent susceptibility of the kidney to the carcinogen.

The influence of changes in diet, or other treatments, on the metabolism of DMN in various organs can be estimated by measuring the metabolism by tissue slices *in vitro*, or the alkylation of DNA of different organs produced by a dose of DMN *in vivo* (Swann & McLean, 1971). The protein-deficient diet increases the amount of nitrosamine metabolized in the kidney because metabolism is inhibited more in the liver than in the kidney (Swann & McLean, 1971). In these CFN rats the metabolism of DMN by liver slices was reduced to 26% of normal by the protein-free diet, whilst the metabolism by kidney slices was reduced to 56% of normal. The rat excretes only a small amount of DMN, so the selective inhibition of metabolism in the liver increases the proportion of the dose metabolized in the kidney and increases the alkylation of kidney DNA. The amount of alkylation of kidney DNA of rats on the protein-free diet was 1·7 times greater than in the kidneys of rats on a normal diet. If the tumour incidence was related to the amount of alkylation one would expect 23 mg DMN/kg in rats on a protein-free diet to produce the same incidence of tumours as 40 mg/kg on a normal diet. The incidence of tumours at various times in rats given 40 mg DMN/kg on a normal diet (Fig. 1(b)) can be expressed as probits and substituted into the equations describing the dose response in the protein-deprived rats (Results Section). It is then estimated that rats on a protein-free diet require only 24·4 mg DMN/kg to produce the same incidence. (The individual estimates were 23·4, 25·6, 23·8, 25·0, and 24·0 mg/kg when the comparison was made at 50, 60, 70, 80, 90, and 100 weeks.)

Since the relative effectiveness of the carcinogen predicted from the amount of alkylation of kidney DNA produced by DMN in rats on the 2 dietary regimes is so similar to the relative effectiveness observed, it seems that the protein-free diet produces little change in the inherent susceptibility of the kidney to this carcinogen.

Benzo(a)pyrene pre-treatment partially reverses the effect of the protein-free diet on the rate of metabolism of DMN by liver slices, increasing it from 26% to 78% of normal. One might expect therefore that pre-treatment with benzo(a)pyrene would increase the toxicity of the nitrosamine. Although a formal $LD_{50}$ was not carried out, this prediction accords with the observation that whereas 24% of the rats on the protein-free diet were killed by 40 mg DMN/kg, 55% were killed when the rats were pre-treated with benzo(a)-pyrene (Table I).

The metabolic studies suggested that pre-treatment with benzo(a)pyrene should have less effect on the incidence of kidney tumours than it had on toxicity. Treatment with benzo(a)pyrene restores the activity of the liver slices of the protein-deprived rat to 78% of normal, but increases the activity of kidney slices to 115% of normal. Thus although the overall rate of metabolism had been largely restored, the balance is towards metabolism by the kidney. The alkylation of kidney DNA by 40 mg DMN/kg was the same in the benzo(a)pyrene-treated, protein-deprived rats, as in those on the protein-free diet alone (0·12 μmol N-7-methylguanine/mol guanine). Thus if tumour incidence were related to the amount of alkylation one would not expect benzo(a)pyrene treatment to affect the kidney-tumour incidence produced by DMN in the protein-deprived rat. The results (Fig. 1(a)) are consistent with that prediction. The incidence of tumours at various times in the rats given 40 mg DMN after treatment with benzo(a)pyrene can be converted to probits and substituted in the equations given in the results. It is then found that the apparent median tumorigenic effect is that expected from 37·1 mg DMN in the untreated rat (the individual values are 35·7, 39·4, 39·7, 39·9, 36·8 and 34·0 mg/kg at 50, 60, 70, 80, 90 and 100 weeks).

These results are preliminary, and further work is needed on the relationship between the metabolism of DMN, the alkylation of nucleic acids, and the carcinogenic activity of the compound. These results suggest, however, that when a given treatment changes the incidence of tumours, it should be possible to assess how much that change is due to a change in the metabolism of the introsamine and how much it is due to a change in the inherent susceptibility of the tissue.

We are most grateful to Laurence Freedman for his help and advice with the mathematics, and to Professor H. Druckrey for his interest and encouragement. This project has been most generously supported by the Cancer Research Campaign.

### REFERENCES

BARNES, J. M. & MAGEE, P. N. (1954) Some toxic properties of dimethylnitrosamine. *Br. J. Indust. Med.*, **11**, 167.

BARRETT, J. C. & TS'O, P. O. P. (1978) Evidence for the progressive nature of neoplastic transformation *in vitro*. *Proc. Natl Acad. Sci., U.S.A.*, **75**, 3761.

CRADDOCK, V. M. (1976) Cell proliferation and experimental liver cancer. In *Liver Cell Cancer*, H. M. Cameron *et al.* (Eds). Amsterdam: Elsevier. p. 153.

DOLL, R. (1971) The age distribution of cancer: Implications for models of carcinogenesis (with discussion). *J. R. Stat. Soc., Series A*, **134**, 133.

DRUCKREY, H., STEINHOFF, D., PREUSSMANN, R. & IVANCOVIC, S. (1964) Erzeugung von Krebs durch eine einmalige Dosis von Methyl-nitroso-harnstoffs und verschiedenen Dialkylnitrosaminen an Ratten. *Z. Krebsforsch.*, **66**, 1.

DRUCKREY, H. (1967) Quantitative aspects in chemical carcinogenesis. In *Potential Carcinogenic Hazard from Drugs: Evaluation of Risks*, Ed. R. Truhaut. Berlin: Springer. p. 60.

DRUCKREY, H., SCHLAGEN, B. & IVANCOVIC, S. (1970) Erzeugung neurogener Malignome durch einmalige Gabe von Athyl-nitrosoharnstoff (ANH) an neugeborene und junge BD IX-Ratten. *Z. Krebsforsch.*, **74**, 141.

DUTTON, A. H. & HEATH, D. F. (1956) The preparation of [$^{14}$C]dimethylamine and [$^{14}$C]dimethylnitrosamine. *J. Chem. Soc.*, 1892.

FINNEY, D. J. (1971) *Probit Analysis*, 3rd Edn. Cambridge: University Press.

FOULDS, L. (1969) *Neoplastic Development*, Vol. 1. London: Academic Press.

HARD, G. C. (1978) Histological conformity of implantation tumours produced by kidney cell lines derived from dimethylnitrosamine-treated rats with dimethylnitrosamine-induced renal mesenchymal tumours. *Cancer Res.*, **38**, 1974.

HARD, G. C. & BORLAND, R. (1977) Morphologic character of transforming renal cell cultures derived from Wistar rats given dimethylnitrosamine. *J. Natl Cancer Inst.*, **58**, 1377.

HARD, G. C. & BUTLER, W. H. (1970) Cellular analysis of renal neoplasia: Induction of renal tumours in dietary conditioned rats by dimethylnitrosamine with a reappraisal of morphological characteristics. *Cancer Res.*, **30**, 2796.

HARD, G. C. & BUTLER, W. H. (1971) Ultrastructural study of the development of interstitial lesions leading to mesenchymal neoplasia induced in the rat renal cortex by dimethylnitrosamine. *Cancer Res.*, **31**, 337.

LAERUM, O. D. & RAJEWSKY, M. (1975) Neoplastic transformation of fetal rat brain cells in culture after exposure to ethylnitrosourea *in vivo*. *J. Natl Cancer Inst.*, **55**, 1177.

MAGEE, P. N. & BARNES, J. M. (1962) Induction of kidney tumours in the rat with dimethylnitrosamine (N-nitroso-dimethylamine). *J. Pathol. Bacteriol.*, **84**, 19.

MCLEAN, A. E. M. & MCLEAN, E. K. (1966) The effect of diet and 1,1,1-trichloro-2,2-bis-(p-chlorophenyl)ethane (DDT) on microsomal hydroxylating enzymes and on the sensitivity of rats to carbon tetrachloride poisoning. *Biochem. J.*, **100**, 564.

MCLEAN, A. E. M. & VERSCHUUREN, H. G. (1969) Effects of diet and microsomal enzyme induction on the toxicity of dimethyl nitrosamine. *Br. J. Exp. Pathol.*, **50**, 22.

McLean, A. E. M. & Magee, P. N. (1970) Increased renal carcinogenesis by dimethylnitrosamine in protein deficient rats. *Br. J. Exp. Pathol.*, **51**, 587.

Peto, R. (1977) Epidemiology, multi stage models, and short term mutagenicity tests. In *The Origins of Human Cancer*, Book C. Eds H. H. Hiatt *et al.* Cold Spring Harbor Laboratory. p. 1403.

Roscoe, J. P. & Claisse, P. J. (1978) Analysis of N-ethyl-N-nitrosourea-induced brain carcinogenesis by sequential culturing during the latent period. I. Morphology and tumorigenicity of the cultured cells and their growth in agar. *J. Natl Cancer Inst.*, **61**, 381.

Stewart, B. W. & Magee, P. N. (1973) Modification of dimethylnitrosamine-induced changes in renal metabolism and subsequent effect on carcinogenic activity of actinomycin D and cycloheximide. *Eur. J. Cancer*, **9**, 37.

Swann, P. F. & McLean, A. E. M. (1968) The effect of diet on the toxic and carcinogenic action of dimethylnitrosamine. *Biochem. J.*, **107**, 14p.

Swann, P. F. & McLean, A. E. M. (1971) Cellular injury and carcinogenesis. The effect of a protein free, high carbohydrate diet on the metabolism of dimethylnitrosamine in the rat. *Biochem. J.*, **124**, 283.

Swenberg, J. A., Koestner, A., Wechsler, W. & Denlinger, R. H. (1972) Quantitative aspects of transplacental tumor induction with ethylnitrosourea in rats. *Cancer Res.*, **32**, 2656.

Waynforth, H. B., Parkin, R. & Stoddart, D. J. (1977) The effect of a protein-free diet, a sugar diet and of carbon tetrachloride administration on the toxicity and rate of metabolism of dimethylnitrosamine in different rat strains. *Br. J. Exp. Pathol.*, **58**, 225.

Weil, C. S. (1952) Tables for convenient calculation of median effective dose ($LD_{50}$ or $ED_{50}$) and instructions in their use. *Biometrics*, **8**, 249.

Wodinsky, I., Helinski, A. & Kensler, C. J. (1965) Experimental tumorigenesis in the hamster cheek pouch. *Nature*, **207**, 770.


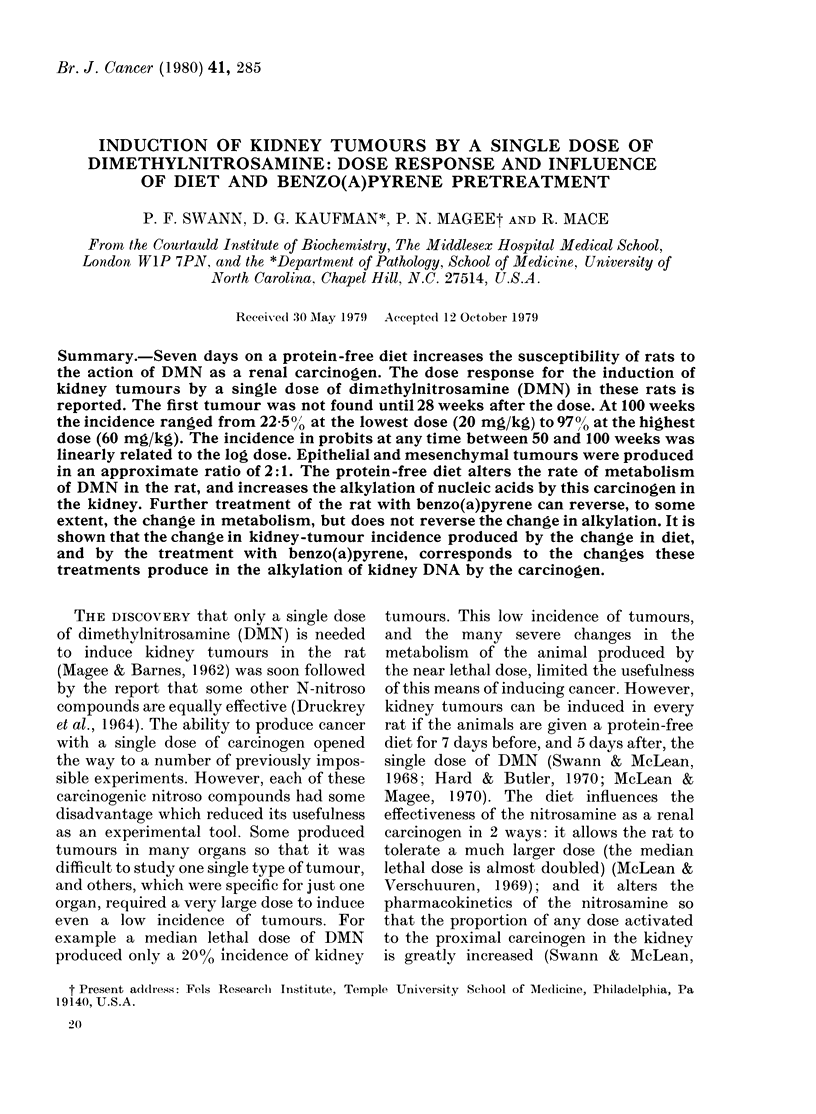

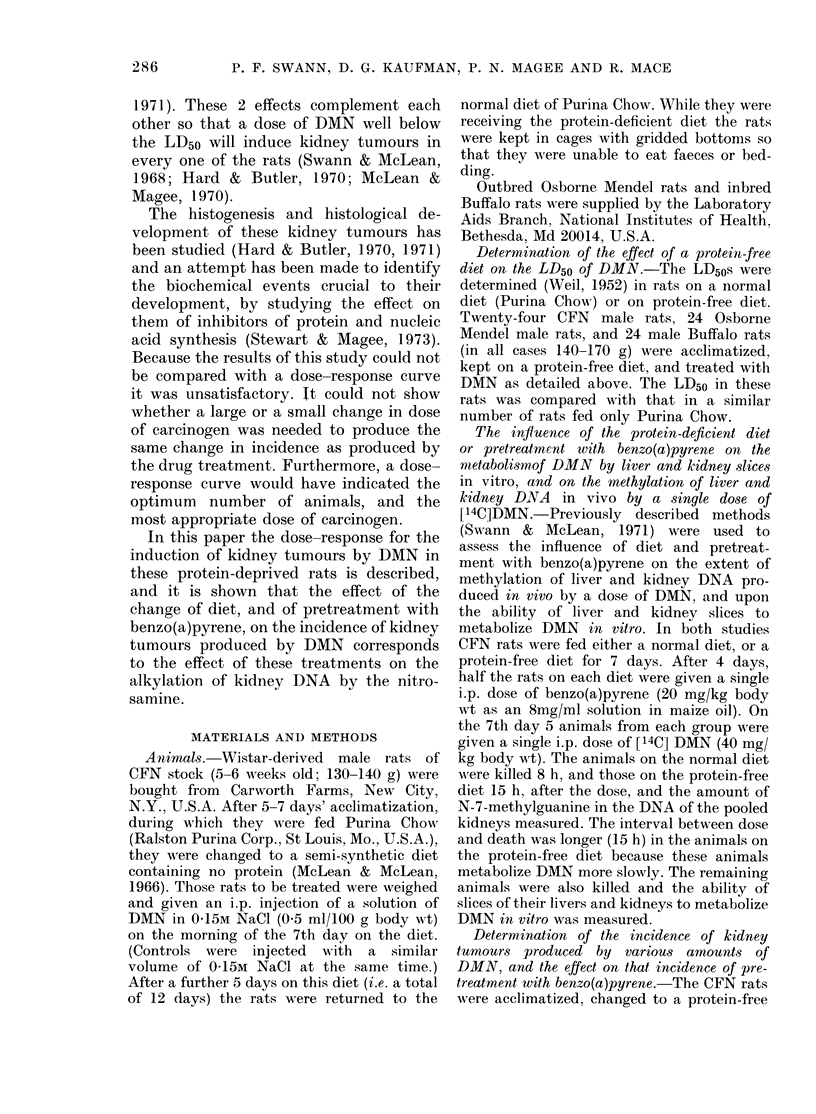

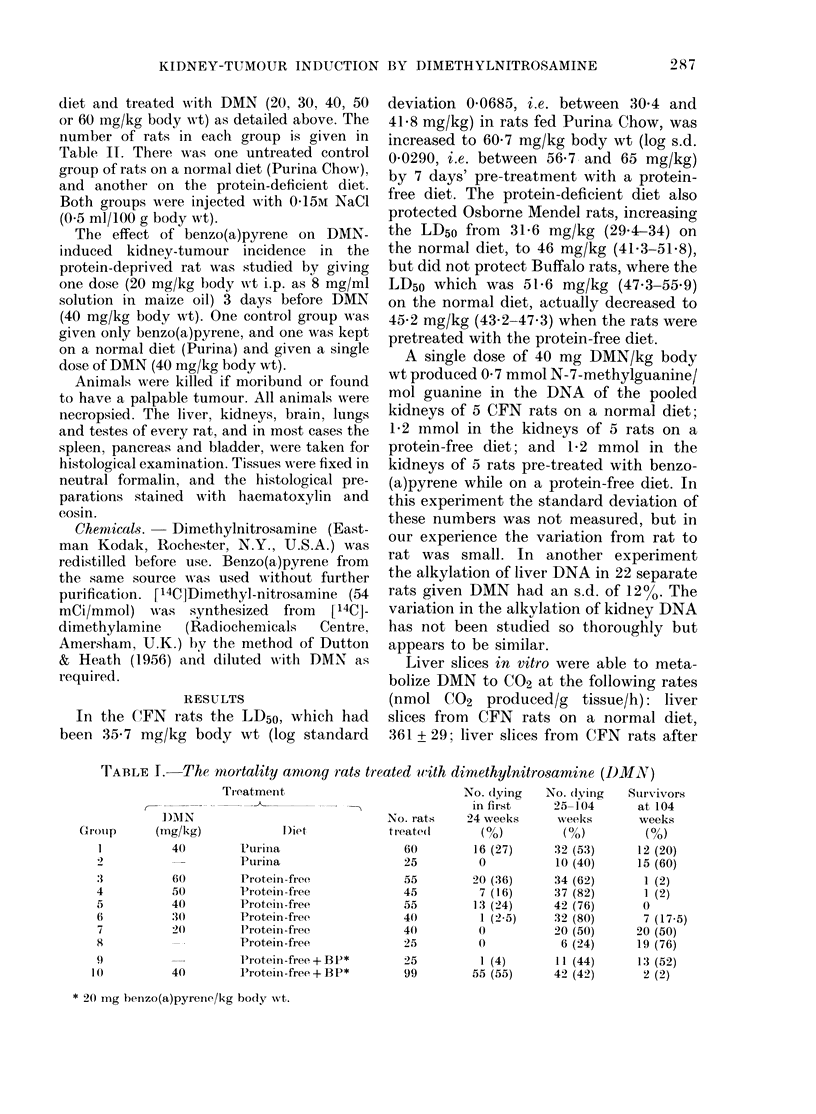

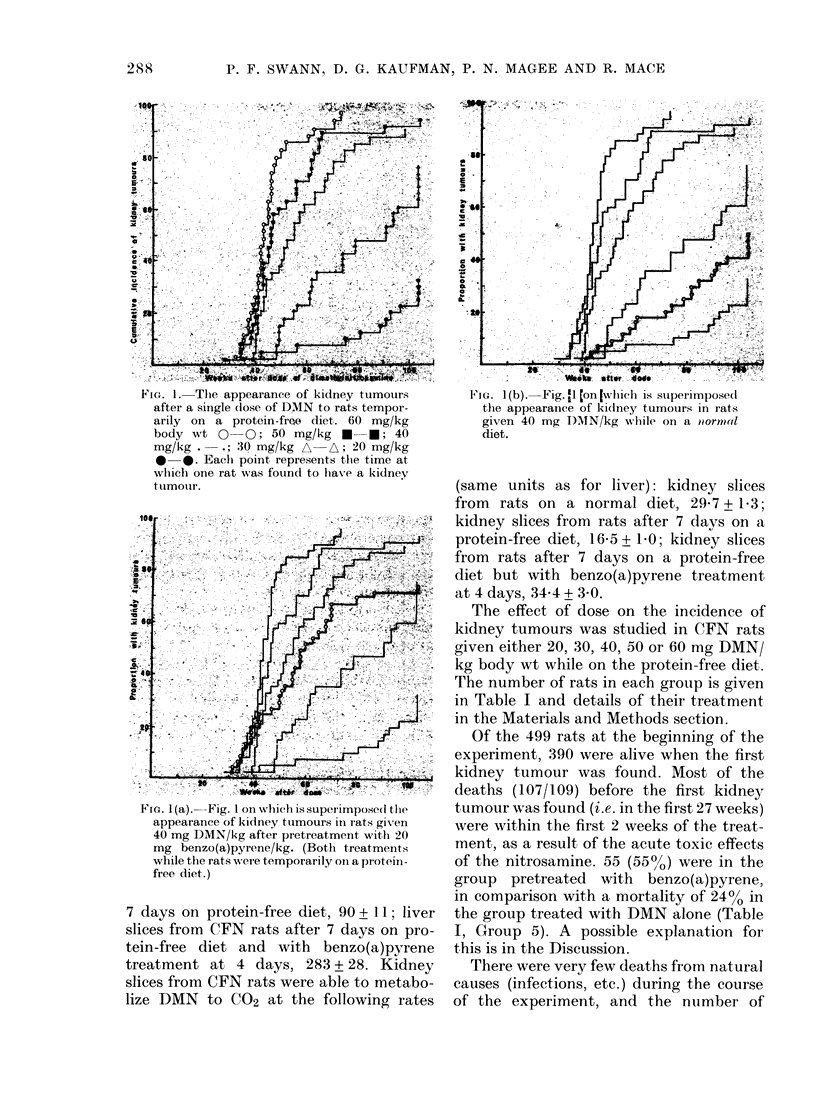

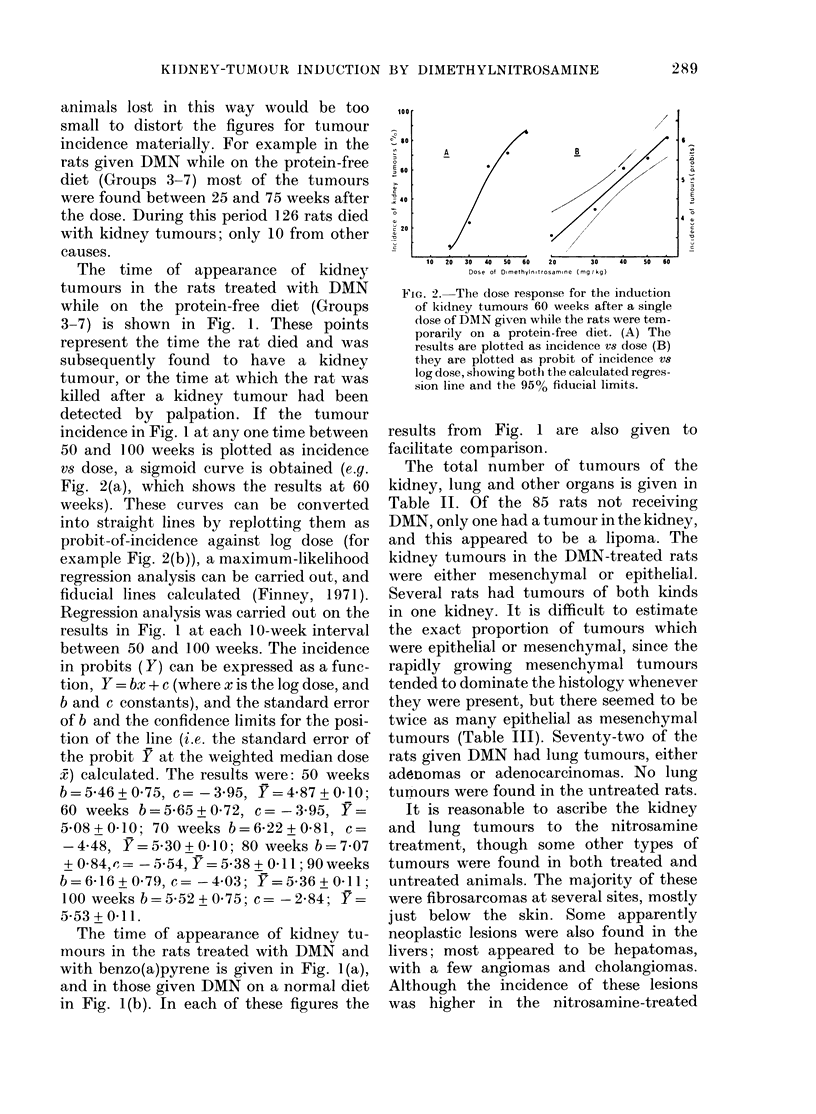

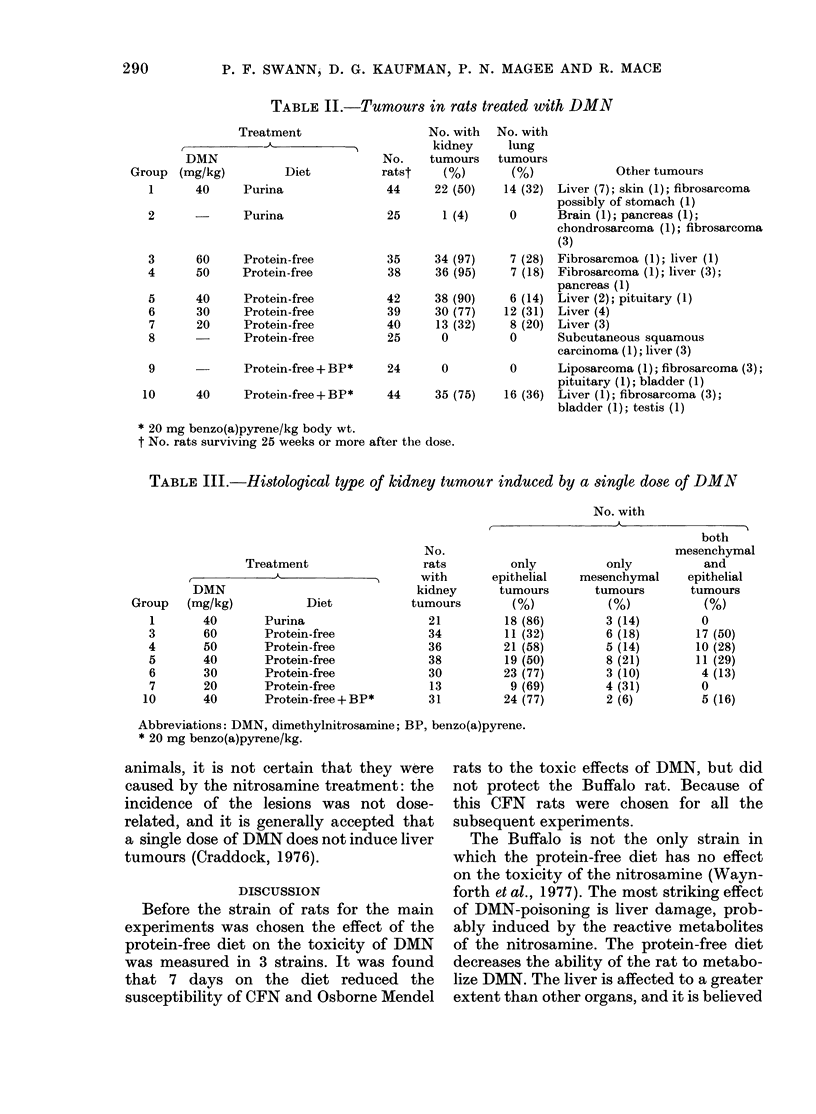

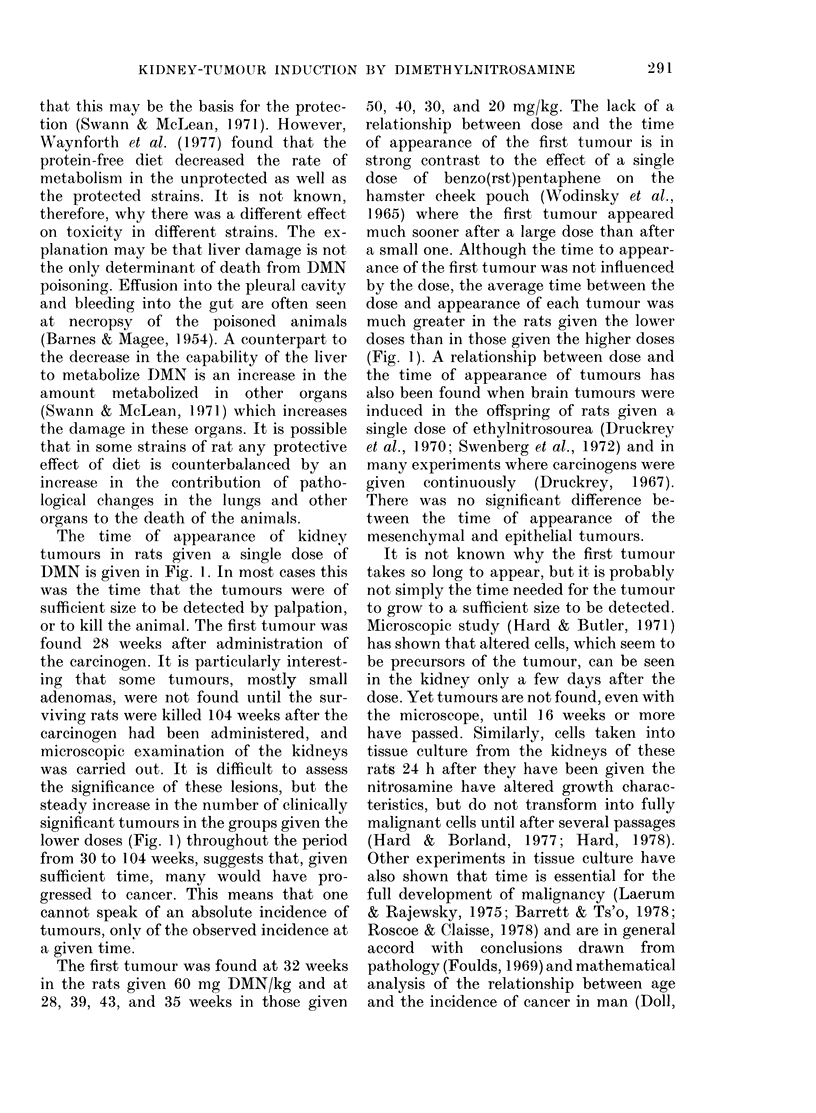

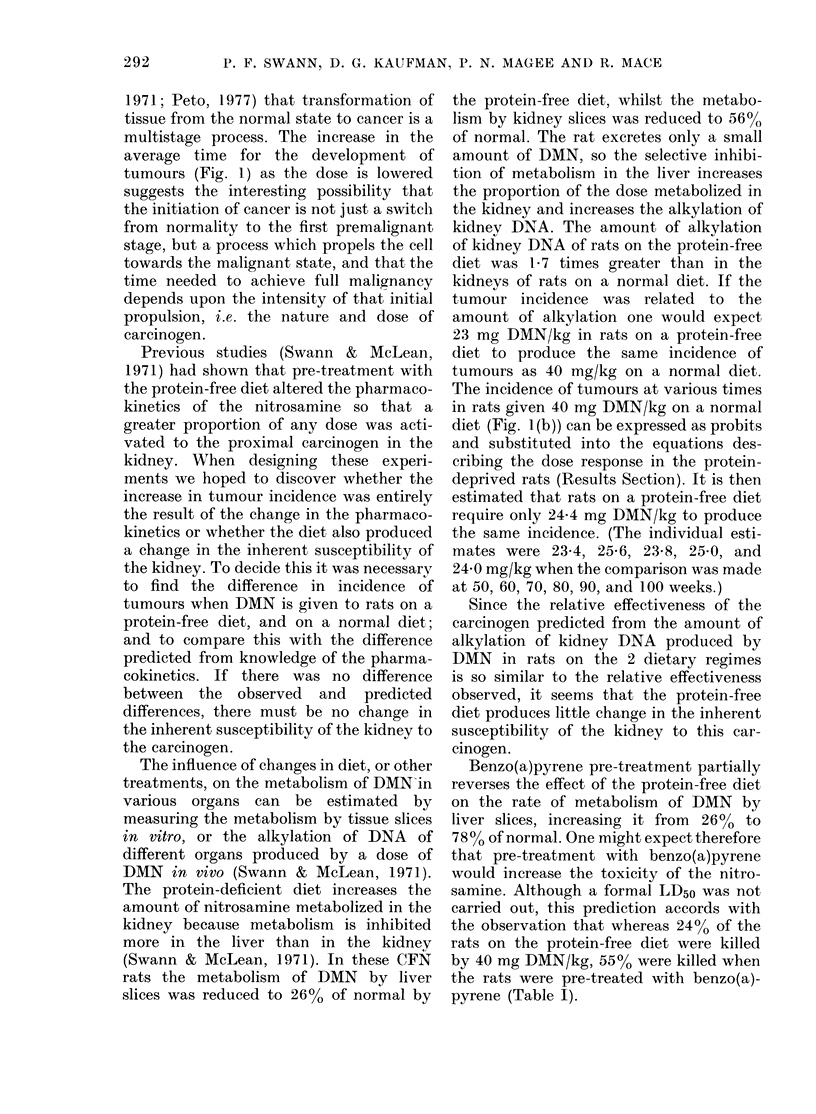

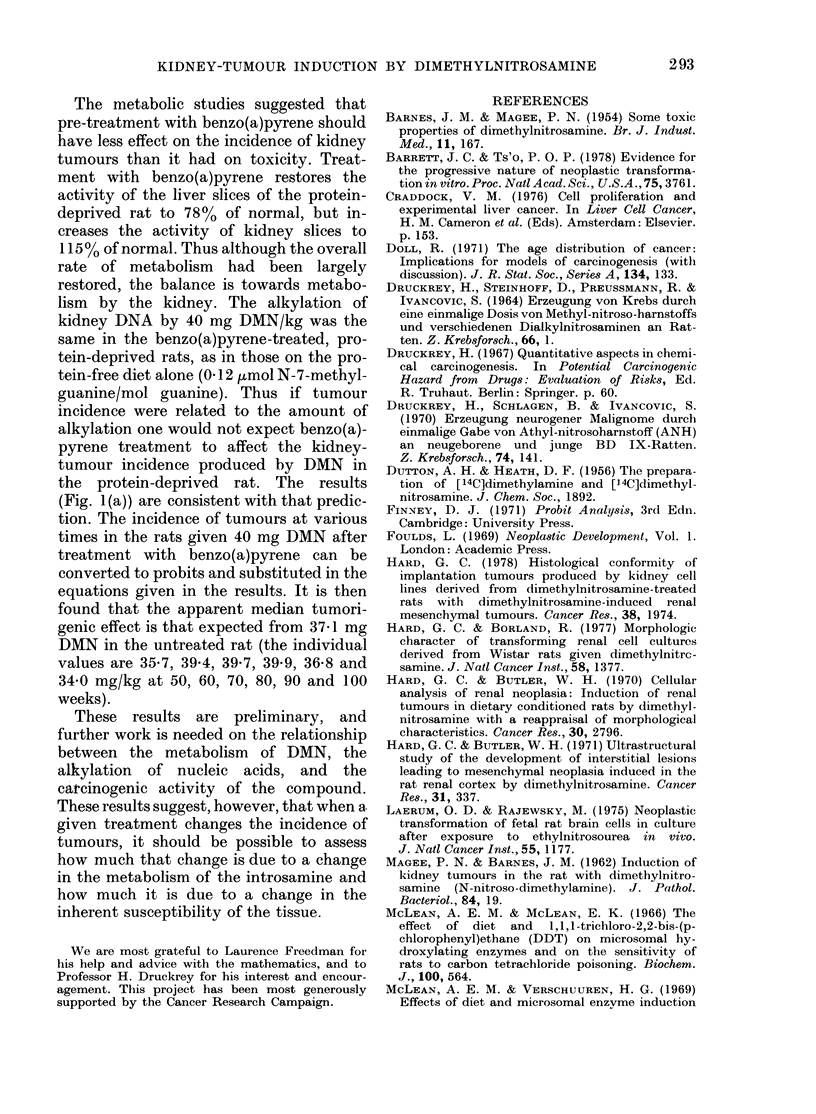

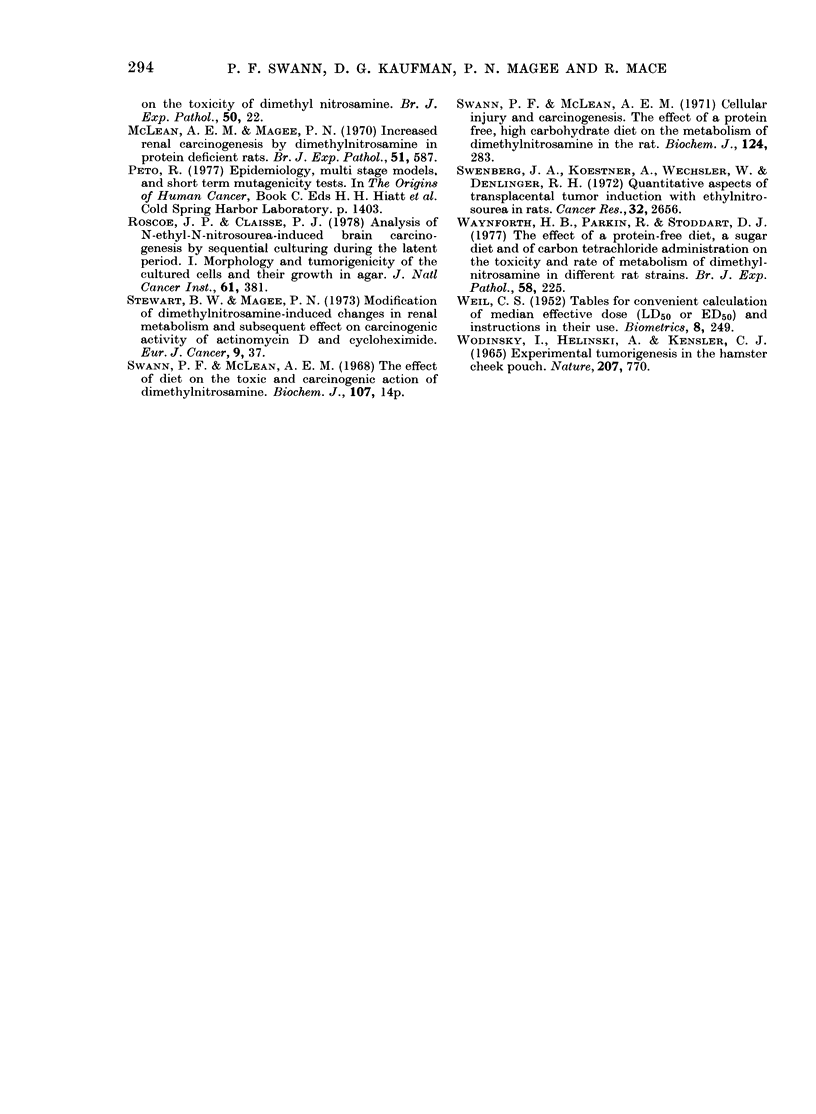


## References

[OCR_01122] BARNES J. M., MAGEE P. N. (1954). Some toxic properties of dimethylnitrosamine.. Br J Ind Med.

[OCR_01127] Barrett J. C., Ts'o P. O. (1978). Evidence for the progressive nature of neoplastic transformation in vitro.. Proc Natl Acad Sci U S A.

[OCR_01142] DRUCKREY H., STEINHOFF D., PREUSSMANN R., IVANKOVIC S. (1964). ERZEUGUNG VON KREBS DURCH EINE EINMALIGE DOSIS VON METHYLNITROSO-HARNSTOFF UND VERSCHIEDENEN DIALKYLNITROSAMINEN AN RATTEN.. Z Krebsforsch.

[OCR_01155] Druckrey H., Schagen B., Ivankovic S. (1970). Erzeugung neurogener Malignome durch einmalige Gabe von Athyl-nitrosoharnstoff (ANH) an neugeborene und junge BD IX-Ratten.. Z Krebsforsch.

[OCR_01182] Hard G. C., Borland R. (1977). Morphologic character of transforming renal cell cultures derived from Wistar rats given dimethylnitrosamine.. J Natl Cancer Inst.

[OCR_01188] Hard G. C., Butler W. H. (1970). Cellular analysis of renal neoplasia: induction of renal tumors in dietary-conditioned rats by dimethylnitrosamine, with a reappraisal of morphological characteristics.. Cancer Res.

[OCR_01195] Hard G. C., Butler W. H. (1971). Ultrastructural study of the development of interstitial lesions leading to mesenchymal neoplasia induced in the rat renal cortex by dimethylnitrosamine.. Cancer Res.

[OCR_01175] Hard G. C. (1978). Histological conformity of implantation tumors produced by kidney cell lines derived from dimethylnitrosamine-treated rats, with dimethylnitrosamine-induced renal mesenchymal tumors.. Cancer Res.

[OCR_01202] Laerum O. D., Rajewsky M. F. (1975). Neoplastic transformation of fetal rat brain cells in culture after exposure to ethylnitrosourea in vivo.. J Natl Cancer Inst.

[OCR_01208] MAGEE P. N., BARNES J. M. (1962). Induction of kidney tumours in the rat with dimethylnitrosamine (N-nitrosodimethylamine).. J Pathol Bacteriol.

[OCR_01231] McLean A. E., Magee P. N. (1970). Increased renal carcinogenesis by dimethyl nitrosamine in protein deficient rats.. Br J Exp Pathol.

[OCR_01214] McLean A. E., McLean E. K. (1966). The effect of diet and 1,1,1-trichloro-2,2-bis-(p-chlorophenyl)ethane (DDT) on microsomal hydroxylating enzymes and on sensitivity of rats to carbon tetrachloride poisoning.. Biochem J.

[OCR_01222] McLean A. E., Verschuuren H. G. (1969). Effects of diet and microsomal enzyme induction on the toxicity of dimethyl nitrosamine.. Br J Exp Pathol.

[OCR_01241] Roscoe J. P., Claisse P. J. (1978). Analysis of N-ethyl-N-nitrosourea-induced brain carcinogenesis by sequential culturing during the latent period. I. Morphology and tumorigenicity of the cultured cells and their growth in agar.. J Natl Cancer Inst.

[OCR_01249] Stewart B. W., Magee P. N. (1973). Modification of dimethylnitrosamine-induced changes in renal metabolism and subsequent effect on carcinogenic activity of actinomycin D and cycloheximide.. Eur J Cancer.

[OCR_01261] Swann P. F., McLean A. E. (1971). Cellular injury and carcinogenesis. The effect of a protein-free high-carbohydrate diet on the metabolism of dimethylnitrosamine in the rat.. Biochem J.

[OCR_01268] Swenberg J. A., Koestner A., Wechsler W., Denlinger R. H. (1972). Quantitative aspects of transplacental tumor induction with ethylnitrosourea in rats.. Cancer Res.

[OCR_01274] Waynforth H. B., Parkin R., Stoddart D. J. (1977). The effect of a protein-free diet, a sugar diet and of carbon tetrachloride administration on the toxicity and rate of metabolism of dimethylnitrosamine in different rat strains.. Br J Exp Pathol.

[OCR_01287] Wodinsky I., Helinski A., Kensler C. J. (1965). Experimental tumorigenesis in the hamster cheek pouch.. Nature.

